# The Editor of the Medical Record Retires

**Published:** 1904-09

**Authors:** 


### The Editor of the Medical Record Retires.

DR. GEORGE F. SHRADY, the distinguished and learned editor of the *Medical Record,* announced his retirement from the editorial field in his journal for August 6, 1904. This notice came as a surprise to the journalistic world, though doubtless his immediate associates had received some preliminary intimation of his intention to resign.

The career of Dr. Shrady, both editorial and professional, has been an eminent one and for the past thirty-eight years he has kept in touch with the progressive science of medicine and surgery throughout the world, promulgating through the columns of the *Medical Record* whatever of value has been discovered that would serve to make for improvement in medicine and medical education.

Dr. Shrady was singularly fitted by training and education for establishing upon a sound and substantial basis the leading medical weekly in America. It is no disparagement to those influential journals which have been established since the birth of the *Medical Record,* to claim for that periodical and its editor a foremost place in the editorial and journalistic field. He has, indeed, become the dean of the American medical editorial corps.

We cannot forget, among the many excellent causes the *Medical Record* has championed, that Dr. Shrady has ever been the friend and advocate of higher medical education; that during the long struggle to establish state examinations for license the columns of the *Medical Record* bristled with editorial articles supporting the measure; and that, during the later discussions regarding reciprocity and medical licensure, some of which were quite sophomoric in their attacks on the system prevailing in this state, Dr. Shrady's pen was wielded in favor of maintaining the New York standard.

It has been the privilege of the editor of this journal to enjoy Dr. Shrady's personal acquaintance for twenty-five years, and we feel that his retirement is in a degree a personal loss. The JOURNAL, however, extends its congratulations upon his release from the perturbations of editorial life and trusts that his years may be many in this land of peace and plenty; and, further, that he may continue to lend his powerful influence in favor of all that goes to make the American medical profession stronger and better.

